# Bilateral Cerebral Calcifications in Secondary Fahr’s Syndrome

**DOI:** 10.1155/crnm/5598992

**Published:** 2025-12-20

**Authors:** Andreas Posa, Stefan Schob, Walter A. Wohlgemuth, Malte E. Kornhuber

**Affiliations:** ^1^ University Clinic and Outpatient Clinic for Radiology, Martin Luther University Halle-Wittenberg, Halle, Germany, uni-halle.de; ^2^ University Clinic and Outpatient Clinic for Neurology, Martin Luther University Halle-Wittenberg, Halle, Germany, uni-halle.de; ^3^ Neurological Clinic, HELIOS Hospital Sangerhausen, Sangerhausen, Germany

**Keywords:** bilateral cerebral calcification, Fahr’s disease, hypocalcaemia, hypoparathyroidism, secondary Fahr’s syndrome

## Abstract

Fahr’s syndrome is a rare, slowly progressive neurodegenerative disorder characterised by bilateral cerebral calcifications, mostly in the basal ganglia. These cerebral calcifications are composed of calcium and phosphate and are the result of disturbances in calcium‐phosphate homeostasis. The clinical manifestations include neurological, neurocognitive and psychiatric symptoms. This article describes three rare cases of pronounced bilateral cerebral calcifications. All three patients were admitted to the hospital due to a first‐time epileptic seizure. In all three cases, laboratory tests showed significant hypocalcaemia, and cerebral computed tomography showed pronounced bilateral cerebral calcifications in various brain areas. After calcium substitution and anticonvulsant treatment, the patients returned to their prehospital condition and were discharged home seizure free. The aim of this article is to highlight the clinical importance of long‐term follow‐up biochemical laboratory testing and neurocranial imaging in high‐risk patients (e.g., after thyroidectomy) to prevent avoidable neurological and psychiatric complications through pharmaceutical and nutritional substitution therapy.

## 1. Introduction

Fahr’s syndrome (FS) is a rare, slowly progressive neurodegenerative disorder characterised by cerebral calcifications (CC). It is associated with abnormalities in calcium‐phosphate homeostasis (CPH), which lead to an abnormal calcium‐phosphate ratio (CPR) and the precipitation of calcium hydroxyapatite crystals in the tunica media of medium‐ and small‐sized cerebral arteries and capillaries [1–4]. Although the specific mechanism of the link between CC and hypocalcaemia is not clear [3, 5], increased formation of calcium‐phosphate complexes, local disruption of the blood‐brain barrier, loss of pericyte maintenance or disruption of neuronal calcium metabolism appear to be involved in lowering calcium in the brain parenchyma [5–12].

CC can be primary due to genetic mutations (primary familial brain calcification, PFBC) [4, 5, 13–20] or secondary to hypocalcaemia, parathyroid disorders, other metabolic disorders, mitochondrial dysfunction, renal failure, toxins, infection, inflammation, autoimmunity, vascular disease, intracranial haemorrhage (IH) or radiotherapy [1, 5, 13, 15, 21–26]. Diagnosis is made by neurocranial computed tomography (CT) scans or magnetic resonance imaging (MRI) [3, 5] and biochemical laboratory tests (low calcium levels, elevated phosphate levels) [3, 27].

Although FS is a very rare neurodegenerative disorder, it occurs repeatedly, even if the secondary variant could be prevented [3, 13, 28]. The aim of this article is to highlight the clinical importance of long‐term follow‐up biochemical laboratory testing and neurocranial imaging in high‐risk patients (e.g., after thyroidectomy) to prevent avoidable neurological and psychiatric complications through pharmaceutical and nutritional substitution therapy.

## 2. Case Description

### 2.1. Case 1

The patient (female, 80 years) was admitted to hospital after an epileptic seizure (ES). Until then, she lived alone in her own apartment, supported by an outpatient nursing service. The ES was observed by the nursing staff: the patient sat sunken and leaning back in the armchair, with no response to speech, tonic tension in the arms, eyes open and slightly rolled upwards, tongue biting and urination. After a few minutes, the patient was responsive again but slowed and drowsy. No history of ES or CC, not even familial. Pre‐existing conditions included atrial fibrillation (permanent), benign arterial hypertension (Grade 1), chronic kidney disease (Grade 3) and obesity (Grade 2). Previous medications included Amlodipine (5 mg, 1‐0‐0), Apixaban (5 mg, 1‐0‐1), Atorvastatin (10 mg, 0‐0‐1), and Pantoprazole (40 mg, 0‐0‐1). Clinical neurological examination showed a slowed patient with tongue bite (at the tip). There was no evidence of motor or sensory, cranial nerve or reflex deficits. Pupil and oculomotor functions were unremarkable. No meningitic signs. Clinically, there was no evidence of infection or dehydration. The patient was cardiopulmonary stable without further ES.

**Figure 1:**
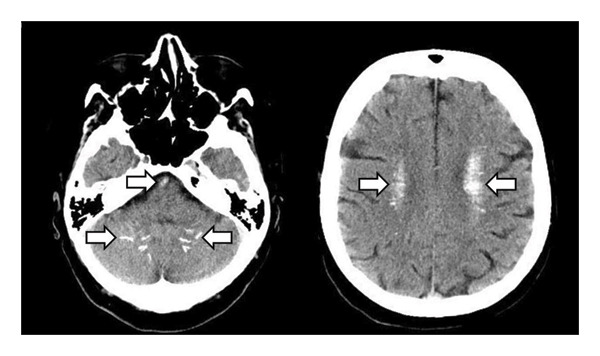
Cranial CT scan (transversal plane). Pronounced cerebral calcifications (arrows) in both cerebellar and cerebral hemispheres in patient 1.

The electroencephalogram (EEG) showed a focus in the left cerebral hemisphere with signs of increased cerebral excitability. A cranial CT showed pronounced CC in both cerebellar and cerebral hemispheres (Figure 1), but no evidence of infarction, IH, severe stenosis or occlusions of cranial vessels (neither intracranial nor extracranial) or cerebrospinal fluid (CSF) circulation disorders. Laboratory tests revealed hypocalcaemia (1.7 mmol/L; norm: 2.2–2.6) and hypoparathyroidism (0.4 pmol/L; norm: 1.6–6.9). Other laboratory values were not clinically significant. No evidence of infection (normal values for C‐reactive protein (CRP) and leukocytes and for urine analysis) or further electrolyte shifts (normal values for sodium and potassium).

In summary, ES was due to a structural genesis with pronounced CC with hypocalcaemia and hypoparathyroidism. CT and laboratory tests did not reveal any competing causes. Substitution therapy with calcium (500 mg, 1‐0‐1), cholecalciferol (1000 IU, 1‐0‐0) and parathyroid hormone (50 μg, 1‐0‐0) and anticonvulsant treatment with levetiracetam (750 mg, 1‐0‐1) were started. The patient remained seizure free and was able to return home in her previous condition two days after admission.

### 2.2. Case 2

The patient (female, 79 years) was admitted to hospital after ES. Previously, she lived independently with her husband in her own apartment, supported by an outpatient nursing service. The ES was observed by the husband: the patient was sitting at the kitchen table to eat, suddenly tipped sideways against the wall and had a tonic‐clonic release of the arms, hyperextended posture, no reaction to speech, eyes open and urination. After a few minutes, she was responsive again, slowed down and drowsy. No history of ES or CC, not even familial. Pre‐existing conditions included atrial fibrillation (permanent), benign arterial hypertension (Grade 1), heart failure (Grade 2), mitral valve insufficiency (Grade 1), atrioventricular block (Grade 1), chronic kidney disease (Grade 4), total thyroidectomy (due to nodular goiter), obesity (Grade 1), cataracts (both eyes) and urge incontinence. Previous medications included Apixaban (2.5 mg, 1‐0‐1), Candesartan (8 mg, 1‐0‐0), Levothyroxine (100 μg, 1‐0‐0), Metoprolol (47.5 mg, 0.5‐0‐0), Simvastatin (40 mg, 0‐0‐1), Solifenacin (5 mg, 0‐0‐1) and Torasemide (10 mg, 1‐1‐0). Clinical neurological examination showed a slowed patient. There was no evidence of motor or sensory, cranial nerve or reflex deficits. Pupil and oculomotor functions were unremarkable. No meningitic signs. Clinically, there was no evidence of infection or dehydration. The patient was cardiopulmonary stable without further ES.

**Figure 2:**
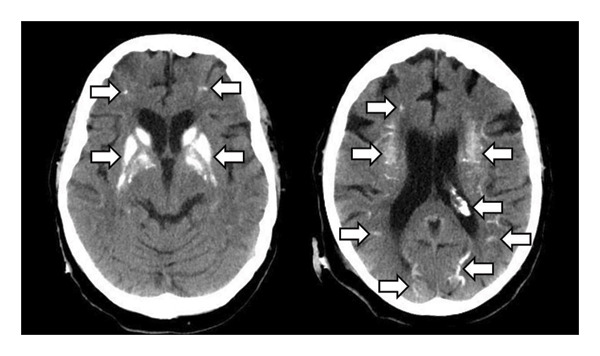
Cranial CT scan (transversal plane). Pronounced cerebral calcifications (arrows) in both basal ganglia, both cerebral hemispheres and choroid plexus in patient 2.

EEG showed a focus in the left cerebral hemisphere with signs of increased cerebral excitability. A cranial CT showed pronounced CC in both basal ganglia, both cerebral hemispheres and in the choroid plexus (Figure 2), but no evidence of infarction, IH, severe stenosis or occlusions of cranial vessels or CSF circulation disorders. Laboratory tests revealed hypocalcaemia (1.5 mmol/L; norm: 2.2–2.6) and hypoparathyroidism (1.1 pmol/L; norm: 1.6–6.9). Other laboratory values were not clinically significant. No indication of infection (normal values for CRP and leukocytes and for urine analysis) or further electrolyte shifts (normal values for sodium and potassium).

In summary, ES was due to a structural genesis, with pronounced CC with hypocalcaemia and hypoparathyroidism. CT and laboratory tests did not reveal any competing causes. Substitution therapy with calcium (500 mg, 1‐0‐1), cholecalciferol (1000 IU, 1‐0‐0) and parathyroid hormone (50 μg, 1‐0‐0) and anticonvulsant treatment with levetiracetam (500 mg, 1‐0‐1) were started. The patient remained seizure free and was able to return home in her previous condition three days after admission.

### 2.3. Case 3

The patient (female, 89 years) was admitted to hospital after ES. Previously, she lived in a nursing home due to dementia. The ES was observed by the nursing staff: the patient was sitting in the wheelchair tilted to the right, with no response to speech, clonic discharge on the right arm, eyes slightly open and tongue biting. Due to diaper use, there was no statement on ES‐related urination. The patient was responsive again after a few minutes but noticeably slowed down and drowsy. No history of ES or CC, not even familial. Pre‐existing conditions included atrial fibrillation (permanent), benign arterial hypertension (Grade 1), chronic kidney disease (Grade 2), heart failure (Grade 2) and dementia. Previous medications included Apixaban (5 mg, 1‐0‐1), and Bisoprolol (1.25 mg, 1‐0‐0). Clinical neurological examination showed a slowed patient with a tongue bite (right lateral). There was no evidence of motor or sensory, cranial nerve or reflex deficits. Due to a pre‐existing dementia, the patient was not orientated to time, place and situation. Pupil and oculomotor functions were unremarkable with no meningitis signs. The patient was cardiopulmonary stable without further ES.

**Figure 3:**
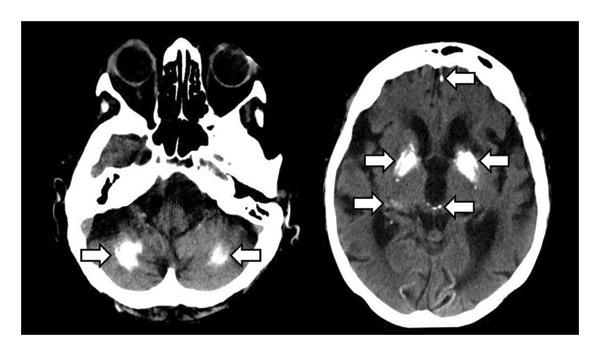
Cranial CT scan (transversal plane). Pronounced cerebral calcifications (arrows) in both basal ganglia and both cerebellar hemispheres in patient 3.

EEG showed a focus in the left cerebral hemisphere with signs of increased cerebral excitability. A cranial CT showed pronounced CC in both basal ganglia and both cerebellar hemispheres (Figure 3). The CT also showed moderate, internally accentuated brain atrophy, but no evidence of infarction, IH, severe stenosis or occlusions of cranial vessels or CSF circulation disorders. Laboratory tests revealed hypocalcaemia (1.8 mmol/L; norm: 2.2–2.6), but parathyroid hormone levels were normal (3.3 pmol/L; norm: 1.6–6.9). In addition, there was a nitrite‐positive urinary tract infection (detection of Escherichia coli), with increased inflammation values (CRP 184.4 mg/L; norm: 0.0–5.0). Other laboratory values were not clinically significant. No indication of further electrolyte shifts (normal values for sodium and potassium).

In summary, ES was due to a structural genesis, with pronounced CC with hypocalcaemia, possibly triggered by a urinary tract infection. CT and laboratory tests revealed no other competing causes for ES. Parathyroid hormone was normal in this case. Substitution therapy with calcium (500 mg, 1‐0‐1) and cholecalciferol (1000 IU, 1‐0‐0), antibiotic therapy with ciprofloxacin (500 mg, 1‐0‐1) and anticonvulsive treatment with levetiracetam (500 mg, 1‐0‐1) were started. The patient remained seizure free and was able to return to the nursing home in her previous condition four days after admission.

## 3. Discussion

Primary Fahr’s disease (FD), or PFBC, is characterised by abnormal deposits of calcium hydroxyapatite crystals in the brain through autosomal dominant genetic mutations in solute carrier family 20 (phosphate transporter) member 2—*SLC20A2* (40% [19]–60% [4]), in platelet‐derived growth factor subunit B—*PDGFB* (∼12%) [4, 16], in platelet‐derived growth factor subunit receptor B—*PDGFRB* (2% [18]–5% [4]) and in xenotropic and polytropic retrovirus receptor 1—*XPR1* (2% [13]–6% [4]) and rarely autosomal recessive (myogenesis regulating glycosidase—*MYORG* (∼13%) [4], junctional adhesion molecule 2—*JAM2* (∼2%) [4] and N‐alpha‐acetyltransferase 60—*NAA60*) [4, 5, 28], which contribute to CC through disrupted CPH, blood‐brain barrier and pericyte function [4, 5, 15, 16, 18]. Peters et al. describe in detail the physiological functions and the pathophysiological mutation consequences of these gene regions [5]. In a recent review, Magrinelli et al. describe the clinical and neuroradiological findings and, importantly, the possible differential diagnoses in FD/PFBC [4]. Secondary FS may be associated with secondary causes such as hypoparathyroidism (e.g., after thyroidectomy) [3, 13, 29–31], which leads to abnormal CPH, resulting in CC [32]. Hypoparathyroidism is one of the important risk factors for secondary FS [3, 5].

Based on the anamnestic, clinical, radiological and laboratory findings, all three patients were diagnosed with FS. In genetic FD, clinical symptoms usually appear between the ages of 30 and 60. An asymptomatic course into old age, as described here, is unlikely in FD. Even with incomplete penetrance, many mutation carriers exhibit at least mild symptoms. Furthermore, FD is usually, though not exclusively, inherited in an autosomal dominant manner. Given the unremarkable family history in all three patients, FD is unlikely. Furthermore, a sufficiently corresponding metabolic explanation for the presence of a suitable secondary FS was found. Given this, further genetic analysis for CC‐associated gene variations was omitted.

Diagnostic criteria of FD and FS have been proposed by Perugula et al. [33] and Saleem et al. [28] and summarised by Jihwaprani and Kumara [3]. CC can be seen as areas of high density on CT [4, 5]. MRI is somewhat inferior to CT because it is less effective at detecting early stages [5] and is less able to distinguish calcium from, e.g., iron deposition in neurodegenerations [4, 34, 35]. Calcium is a diamagnetic substance and has a very low magnetic susceptibility compared with surrounding tissue [4]. Iron, copper and manganese can have similar appearances based on MR sequences used, although MR susceptibility‐weighted imaging filtered phase images may help to distinguish paramagnetic blood products from diamagnetic calcifications [4]. Interestingly, iron [5] and amyloid‐β [30] can also be detected in CC in histopathological studies.

The prevalence of bilateral CC of the basal ganglia observed in neuroimaging is approximately 1% in young patients and > 20% in the elderly [13, 36–39]. CC occur mainly bilaterally and in the basal ganglia (73.8%) [6], but can also affect other brain regions (globus pallidus: 68.8%, putamen: 55.9%, caudate nucleus: 54.8%, grey‐white junction: 39.8%, cerebellar parenchyma: 31.2%, thalamus: 29.0%, dentate nuclei: 24.7%) [6, 15, 31, 40]. Currently, there is no evidence that the localisation or extension of calcification is associated with the type and severity of clinical manifestations [4].

CC can cause a variety of neuropsychological symptoms depending on their intracerebral location [3, 7, 13, 25, 28, 30, 41, 42]. 33% to 50% of FS patients have neurological symptoms [1]. All CC patients presented here suffered from ES. ES is described in 46% of FS patients due to hypoparathyroidism [43]. The clinical presentation and age at presentation are highly variable [44]. This point makes early and correct assignment to FS difficult. The age at onset is mostly between 40 and 60 years [13, 45], but the symptoms can manifest at any age [46, 47]. The severity of CC is correlated with an increase in age, and more calcifications were found in symptomatic patients in comparison with asymptomatic patients [19, 48]. However, studies have not shown a correlation between the amount of CC and the severity of the disease [1]. Interestingly, CC can occur even when the untreated shift in CPH is asymptomatic and subclinical [1, 5, 13, 32, 49]. Thus, it may therefore take years to diagnose [29, 30]. The aim of this article is to raise awareness of this delay in diagnosis in clinical practice and to consider potential risk factors for CC at an early stage.

CC in hypoparathyroidism [22, 50] are related to the duration of hypocalcaemia and hyperphosphatemia (abnormally low CPR) [3, 6], not to parathyroid hormone itself [15, 27], as in patient 3 with a normal parathyroid hormone level. In this case, ES may have been triggered by a urinary tract infection. A previous case report describes the occurrence of neurological symptoms triggered by a respiratory infection [13]. Zavatta et al. showed that a lower CPR is significantly associated with a higher incidence of basal ganglia calcifications [51]. The increased bioavailability of phosphate in the extracellular space can promote the formation of calcium‐phosphate crystals in the brain [51]. For every 1% increase in the CPR, the probability of basal ganglia calcification decreases by 5% [6]. Rarely, CC has been described in hypercalcaemia [2] and hyperparathyroidism [2, 52]. The cause of FS may remain unclear in idiopathic CC, especially in the elderly [1, 13, 53].

In addition to metabolic disorders, as described here, the following differential diagnoses must be considered in CC: (1) genetic diseases (Aicardi–Goutières syndrome, Cockayne syndrome, Krabbe disease, neurofibromatosis, Sturge–Weber syndrome and tuberous sclerosis); (2) infectious diseases (congenital: cytomegalovirus, human immunodeficiency virus, rubella virus, toxoplasma gondii, Zika virus; acquired: cryptococcus neoformans, mycobacterium tuberculosis and taenia solium); (3) tumour diseases (intra‐axial: astrocytoma, ganglioglioma, medulloblastoma and oligodendroglioma; extra‐axial: craniopharyngioma, germ cell tumours, lipoma, meningioma, pineal tumours; intraventricular: central neurocytoma and ependymoma); (4) inflammatory diseases (lupus erythematosus and sarcoidosis) and (5) intoxications (carbon monoxide and lead) [54]. In all these different pathologies, CC has different locations and aspects, which Saade et al. presents in detail in their review [54].

Although gene therapy approaches are being discussed for primarily genetic FD [5], no causal therapy is yet available [1, 3, 4, 13, 55]. Secondary FS, which is caused by a disturbance in CPH, can be treated with substitution therapy (correction of phosphate, calcium, vitamin D and parathyroid hormone levels) [3, 5–7, 56–60]. Most of the patients are managed via symptomatic support [3, 5, 7, 9, 13, 61–63]. A calcium‐rich and phosphate‐reduced diet should also be considered [13]. This point is particularly important for everyday clinical practice, since secondary FS is subject to potentially preventable/treatable causes and can therefore be prevented with early attention and treatment.

## 4. Conclusion

Bilateral CC is a rare but slowly progressive and complex neurodegenerative complication of altered CPH. The exact pathomechanism is not yet well understood. Early diagnosis and treatment of secondary causes is essential to prevent progression of CC and the associated neuropsychological symptoms. Because CC can develop asymptomatically (e.g., after thyroidectomy) and therefore may not be detected for years, long‐term monitoring and regular neurological imaging (especially CT scans) as well as regular biochemical laboratory tests are indicated for appropriate high‐risk patients. Although the development of secondary FS (e.g., due to hypocalcaemia or hypoparathyroidism) has long been recognised, and there are early detection and treatment options, we still encounter cases in everyday clinical practice. This article therefore aims to prevent such cases by considering risk factors and promoting early diagnosis and treatment.

## Ethics Statement

According to the regulations of the Ethics Committee of the Medical Faculty of the Martin Luther University Halle‐Wittenberg, no specific ethical approval is required for a retrospective, anonymized case presentation.

## Consent

Written informed consent was obtained from each patient for publication of this case reports.

## Disclosure

All authors read and approved the final manuscript for submission.

## Conflicts of Interest

The authors declare no conflicts of interest.

## Author Contributions

All authors equally contributed to the concept, design, and data interpretation. Andreas Posa is the guarantor for this paper.

## Funding

The publication of this report received financial support from the open access publication fund of the Martin Luther University Halle‐Wittenberg. Open Access funding enabled and organized by Projekt DEAL.

## Data Availability

Data sharing is not applicable to this article as no datasets were generated or analysed during the current study.
